# Opioid Considerations for Emergency Practice

**DOI:** 10.5811/westjem.2015.12.29447

**Published:** 2015-12-17

**Authors:** Thomas Terndrup

**Affiliations:** Ohio State University College of Medicine, Department of Emergency Medicine, Columbus, Ohio

On a backdrop of increasingly distressing opioid misuse in our communities, and safety concerns expressed by The Joint Commission and others, emergency physicians are further increasing their utilization of these important agents in our patients.^1^,^2^ Are we selecting the best opioid for our patients? Are we providing the relief they need? And are we doing this safely? We all hope these questions can effectively be answered yes, now and into our futures.

The timely report by Sutter et al.^3^ quantifies the increasing use of opioids in their academic ED, demonstrating an increasing preference for hydromorphone (15% absolute increase in 2 years) over morphine (16% reduction), and a stable smaller proportion receiving fentanyl. The authors speculate that this change was driven by new pharmacy rules and electronic record restrictions on the use of morphine, promulgated in their organization. Additionally, in their practice, equipotent dosing of these agents was not generally performed. Finally, naloxone administration concurrent with the ED encounter after an opioid was documented infrequently (0.2% of total naloxone administrations). Generally, patients are satisfied when timely and effective analgesia is received, but disparities and individual variability in practice are still challenges going forward.^4^,^5^

The Patient Safety Movement Foundation recommends that “all patients receiving IV opioids have continuous … pulse oximetry” and those patients receiving supplemental oxygen have continuous respiratory rate monitoring. At a time when alarm fatigue and ED-crowding are factors which may distract providers from adequate monitoring, excessive monitoring may add additional burdens which may not ultimately benefit overall ED patient safety. Clinical personnel close to the bedside during the known peak action of the drug administered can provide both thoughtful monitoring and the immediate ability to respond.. The authors also point out that naloxone reversal was used ~73% of the time for patients with an increased likelihood of respiratory depression. This indicates that patient selection is important in avoiding adverse effects of opioids. Clearly patients with intoxication, sedation and other medications sedative-hypnotic agents on-board should be vigorously monitored and opioid dosing constrained.^6^ Pre-existing headache, back pain, mental health, and substance use disorders were significantly associated with both additional ED and alcohol- or drug-related encounters (ADEs). The use of schedule II long-acting opioids was strongly associated with ADEs and strong cautions against such practices seem warranted.^7^

It has been well documented that we are using opioids to effectively reduce patient suffering, in many differing clinical conditions.^8^–^10^ Time to effective analgesia is especially important for those with severe pain from whatever source. Efforts to reduce unnecessary administration and use of opioids are appropriate, but they should not constrain our ability to provide necessary relief. This includes short-term ED prescriptions for analgesics, where no clear contribution to subsequent abuse can be shown.^11^,^12^ However, given the recent association between opioid prescription and subsequent recurrent use, we should exercise appropriate cautions and use non-opioids when possible.^13^

Increasingly states have adopted approaches to monitor and provide practitioner feedback opportunities to monitor prescription drug prescribing. Reducing over-consumption, diversion and other misuses of prescription medications is an important goal shared by emergency medicine practitioners.^14^

Providing for life-saving and innovative interventions in the ED and our communities should be strongly considered. Mechanisms and policy for advancing the use of community based reversal of opioid over-doses is life-saving.^15^ The administration of agents (like buprenorphine/naloxone) beginning during ED based care, has recently been shown to be beneficial compared to other strategies for opioid addicted patients.^16^

So it seems we have effective analgesic agents with a good safety margin when used appropriately with safe monitoring. Our ability to utilize them appropriately appears to depend upon early administration, dosage adjustment for patients at risk of adverse effects, appropriate monitoring, and advocating for innovations which will assist in reducing the risk of substance abuse and treatments.
